# Atomic Force Microscopy Imaging in Turbid Liquids: A Promising Tool in Nanomedicine

**DOI:** 10.3390/s20133715

**Published:** 2020-07-02

**Authors:** Michael Leitner, Hannah Seferovic, Sarah Stainer, Boris Buchroithner, Christian H. Schwalb, Alexander Deutschinger, Andreas Ebner

**Affiliations:** 1 Institute of Biophysics, Johannes Kepler University, Gruberstraße 40, 4020 Linz, Austria; michael.leitner_1@jku.at (M.L.); hannah.seferovic@jku.at (H.S.); sarah.stainer@jku.at (S.S.); boris.buchroithner@jku.at (B.B.); 2 GETec Microscopy GmbH, Seestadtstraße 27/Top 27, 1220 Vienna, Austria; chris.schwalb@getec-afm.com; 3 SCL-Sensor.Tech. Fabrication GmbH, Seestadtstraße 27/Top 27, 1220 Vienna, Austria; alexander.deutschinger@sclsensortech.com

**Keywords:** piezoresistive cantilever, self-sensing, self-actuating, electrical readout, platelet, all electric AFM, blood, AFSEM

## Abstract

Tracking of biological and physiological processes on the nanoscale is a central part of the growing field of nanomedicine. Although atomic force microscopy (AFM) is one of the most appropriate techniques in this area, investigations in non-transparent fluids such as human blood are not possible with conventional AFMs due to limitations caused by the optical readout. Here, we show a promising approach based on self-sensing cantilevers (SSC) as a replacement for optical readout in biological AFM imaging. Piezo-resistors, in the form of a Wheatstone bridge, are embedded into the cantilever, whereas two of them are placed at the bending edge. This enables the deflection of the cantilever to be precisely recorded by measuring the changes in resistance. Furthermore, the conventional acoustic or magnetic vibration excitation in intermittent contact mode can be replaced by a thermal excitation using a heating loop. We show further developments of existing approaches enabling stable measurements in turbid liquids. Different readout and excitation methods are compared under various environmental conditions, ranging from dry state to human blood. To demonstrate the applicability of our laser-free bio-AFM for nanomedical research, we have selected the hemostatic process of blood coagulation as well as ultra-flat red blood cells in different turbid fluids. Furthermore, the effects on noise and scanning speed of different media are compared. The technical realization is shown (1) on a conventional optical beam deflection (OBD)-based AFM, where we replaced the optical part by a new SSC nose cone, and (2) on an all-electric AFM, which we adapted for measurements in turbid liquids.

## 1. Introduction

Atomic force microscopy (AFM), invented in the late 1980s, has become an invaluable tool in nanoscience. Its field of application ranges from material to life science. Particularly in the life sciences, the unique possibility of molecular resolution under (almost) physiological conditions is convincing, thus justifying the importance of AFM in the study of physiological processes at the single molecule level. This unique possibility was and still is the basis for many successful research projects in which nature has been followed in its work [1,2,3,4,5], opening the window to physiology and nanomedicine. A prerequisite for all these studies was the possibility to perform investigations in transparent liquids (e.g., physiological buffers). A large number of important questions can be answered by measurements at such conditions, but investigations in physiologically relevant turbid liquids are not possible. Conventional AFMs work on the basis of optical readout of the cantilever deflection. For this purpose, a laser beam is focused on the backside of a reflectively coated cantilever. The position of the reflected beam is recorded by a four-segment photodiode. Although it is possible to vary the wavelength of the laser used from UV to infrared, this detection method still fails in the case of highly turbid and light-scattering physiological fluids, such as blood or milk.

The first successes in overcoming the disadvantages caused by optical beam deflection (OBD) were already achieved by Tortonese and colleagues as early as the beginning of the 1990s by implementing a p-type resistor on the surface of a cantilever [6]. Atomic resolution could be achieved by measuring the change in resistance caused by cantilever deflection [7]. Besides the piezoresistive readout based on doped silicon and polysilicon piezo-resistors [7,8,9,10,11,12,13,14,15,16], several other readout methods have been developed in the past to replace the classic OBD readout, such as: capacitive [17,18,19,20,21], piezoelectric [22,23,24,25,26], thin film metals [27] and tuning forks [28,29,30,31]. The installation of strain sensors directly into the cantilever is of particular interest as it offers several advantages over techniques with external readout. Some of the most important advantages are: (i) extremely small cantilevers far below the optical diffraction limit to increase sensitivity and so imaging speed can be realized [32,33], (ii) avoiding interference with photosensitive samples, (iii) the possibility of multi-cantilever arrays [34,35,36] and (iv) combining AFM with other techniques, such as scanning electron microscopy (SEM) [37,38,39,40]. In addition to the new readout method, advances in microfabrication have allowed the direct integration of actuators on the cantilever, such as piezoelectric excitation [41], Lorentz excitation [42], magnetic excitation [43,44,45,46] and thermal excitation [47]. Direct vibration excitation of the cantilever eliminates the need for an external piezo actuator and increases precision and excitation speed [48,49]. Efforts over the last 20 years to further optimize strain sensor readout have resulted in piezoresistive cantilevers that exceed standard optical beam readout in terms of low-noise imaging [15]. Furthermore, a large number of different applications in the AFM field [35,37,38,40,50,51,52], but also for other cantilever sensor techniques, such as torque magnetometry [53] or gas sensors [54], have been published. Although there is a great potential for the use of self-sensing AFM cantilevers in bio-applications, only very few attempts have been documented to use them in liquid [55] or for imaging biological samples in liquid [56]. To the best of our knowledge, biological AFM imaging in turbid physiological liquids has never been shown before.

In this study, we focused on the realization to perform self-sensing cantilevers (SSC)-based AFM imaging of highly interesting biological samples and to test and compare the performance with respect to different environmental and physical conditions. This was realized by upgrading a conventional AFM and by using an all-electric AFM originally developed for implementation in a SEM. We optimized both instruments for working in liquids. Initial experiments were performed in non-conductive deionized water and subsequently in physiologically relevant fluids, such as blood, blood serum and milk, without passivation of the cantilever. A passivation may change the physical parameters and thus worsen the imaging quality. In the approach used here, the entire cantilever is immersed in a sample beaker, which allows measurements in larger quantities of liquid and greatly simplifies handling compared to measurements in small sample aliquots or droplets.

## 2. Materials and Methods

### 2.1. Optical Beam Deflection Measurements

All OBD measurements were performed on a standard Keysight 5500 SPM2 (Keysight, Santa Rosa, CA, USA). Magnetically coated cantilevers (MAC Lever, Type VII) and magnetic excitation were used for the measurements in magnetic excitation mode (MAC Mode^TM^, Keysight, Santa Rosa, CA, USA). Measurements in intermittent contact mode with acoustic excitation and contact mode measurements were performed using Bruker (Bruker, Billerica, MA, USA) MSCT cantilever E and cantilever D, respectively. This applies to all measurements under ambient conditions as well as to measurements in deionized water.

### 2.2. Self-Sensing Cantilevers

For all piezoresistive readout measurements, self-scanning cantilevers of the type PRSA-L300-F50-Si-PCB (300 × 100 µm) (SCL-Sensor.Tech., Vienna, Austria) were used. These cantilevers are equipped with two active piezo-resistors integrated on the cantilever and two passive piezo-resistors integrated on the chip. These four resistors are connected to a Wheatstone bridge. The resistance was about 1 kOhm each for all cantilevers used. Different supply voltages were used for the bridge depending on whether measurements were made in air or liquid. The standard supply voltage is 2.048 V for the dry state. For measurements in liquid, a supply voltage of 0.51 V was used. The cantilever itself has a size of 300 × 100 µm with a spring constant of 1–15 N/m and a resonance frequency of 30–65 kHz. Its carrier silicon chip is mounted on a Printed Circuit Board (PCB), which in turn is equipped with a 10-pin Kyocera standard connector (Kyocera, Kyoto, Japan) for easy connection to the AFM scanner. For the measurement of biological samples in liquids, cantilevers with spring constants of 1–1.3 N m^−1^ were chosen. In addition, these cantilevers are equipped with a heating loop for thermal excitation. Thermal excitation on the Keysight 5500 SPM2 was performed in magnetic AC (MAC) mode configuration, the power through the heating loop was limited to approximately 0.1 W.

### 2.3. Implementation of the SSC in a Commercial AFM with Optical Beam Deflection

**Figure 1:**
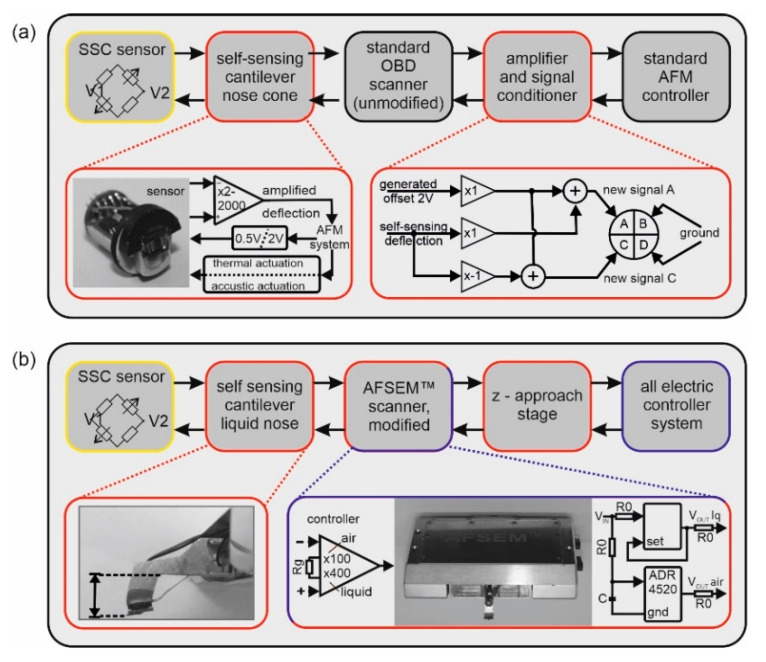
The flow chart in (**a**) shows the essential components for the entire electrical imaging with a commercial atomic force microscope (AFM): The self-sensing cantilever (SSC) (yellow) was mounted on the newly developed nose cone. The self-sensing signal was amplified in the nose cone, passed through an unmodified standard optical beam deflection (OBD) scanner and then subjected to additional signal amplification and processing. This is done in a new hardware between scanner and controller (new hardware components are red, unmodified are black). Part (**b**) of this figure shows a commercial self-sensing cantilever AFM adapted for liquid bio-sensing. The central upgraded parts are the extended nose ((**b**) left) and the new board design for the low-voltage sensor supply ((**b**) right) (new hardware components are red, unmodified ones are blue).

For the implementation of the SSC into a Keysight 5500 SPM2 with OBD, two main technical developments were necessary: (i) a special nose cone (image is shown in Figure 1a left), which on the one hand supports the PCB, including the cantilever, and on the other hand, takes over the bidirectional signal transmission and amplification, and (ii) a second electronic unit between scanner and stage for additional amplification of the deflection signal and to generate signals mimicking a four-quadrant photodiode for the following hardware.

The mechanical part of the nose cone was milled from the high-temperature-resistant thermoplastic polyetheretherketone (PEEK) and adapted to carry the cantilever and the required electronics. The electronics in the nose cone consist of the following components: (i) an instrumentation amplifier with adjustable gain, (ii) power supply for the sensor, switchable between 0.51 V for measurements in liquid and 2.048 V for measurements in air, and (iii) the possibility to switch between thermal excitation via the integrated heating loop or acoustic excitation via a 2 × 2 × 2 mm piezo (P.I., Lederhose, Germany). A multi-layer rigid-flexible PCB combination was developed to connect the electronics inside the nose cone with the sensor placed outside. The nose was designed in such a way that the optical path for positioning the cantilever relative to the sample is still available using the AFM’s CCD camera. This design also ensures that the electronics is as close as possible to the deflection signal’s origin but sealed from the cantilever in liquid. In addition to the voltage supply, the ground signal and the excitation signal (thermal/magnetic or acoustic) are taken from the scanner. The pin, originally intended for conductive measurements, is used for the deflection signal transfer. This configuration ensured that no changes had to be made to the original scanner hardware. The nose is connected to the scanner via the 6 original pins.

The second electronic hardware is located between the scanner, the stage and the photodiode connector and has two tasks: (i) additional deflection signal amplification via an adjustable instrumentation amplifier and (ii) signal conditioning to mimic four-quadrant photodiode signals for the following AFM hardware. For all self-built electronic components, a supply voltage of +/−10 V was taken directly from the photodiode connector. To work with the deflection signal of the self-sensing cantilever, a signal for a four-quadrant photodiode must be imitated for the controller. With optical readout, typically, four signals, A, B, C and D, are obtained. A and B represent the upper part of the four segments of the photodiode and C and D represent the lower part. From these four signals, a single deflection signal is generated using the method given in Equation (1):

(1) $$\frac{\left(A+B\right)-\left(C+D\right)}{A+B+C+D}=normalized\;deflection\;signal$$

The fact that an SSC only emits a single deflection signal means that for the original AFM hardware, a four-quadrant photodiode signal has to be simulated. A scheme for generating the required signals is shown in Figure 1a (right). The original single deflection signal (DS) from the SSC is inverted and an offset voltage (OV) is added to both the original and inverted deflection signal. This results in two new signals (DS + OV) and (−DS + OV). The OV is generated with the same constant voltage source chip that is used for the sensor supply voltage (2.048 V). Now (DS + OV) is used for the photodiode segments A and B, and (−DS + OV) for the segments C and D. Using this in Equation (1) results in:

(2) $$\frac{2*\left(DS+OV\right)-2*\left(-DS+OV\right)}{2*\left(DS+OV\right)+2*\left(-DS+OV\right)}=\frac{DS}{OV}=normalized\;deflection\;signal$$

The following original controller hardware restores the single deflection signal of the SSC. This signal processing is necessary in order to use SSCs on the Keysight 5500 SPM2 AFM without any technical modifications to the original hardware. In the hardware realization, A is set to (DS + OV), C to (−DS + OV), and B and D to ground. This simplifies Equation (2) to:

(3) $$\frac{\left(\left(DS+OV\right)+0\right)-\left(\left(-DS+OV\right)+0\right)\;}{\left(DS+OV\right)+\left(-DS+OV\right)}=\frac{DS}{OV}=normalized\;deflection\;signal$$

All the following components use the normalized single deflection.

### 2.4. Software Implementation

With one exception, the software settings for SSC readout are identical to those for OBD readout: DC offset compensation is performed via the bias input field in the software. If the SSC is located away from the surface, the oscilloscope monitor in the software displays the amplified DC offset. This is compensated via the bias input field, which should result in an oscilloscope signal close to zero. For thermal excitation, the magnetic excitation is selected in the software. Here, the alternating current through the heater excites the cantilever to oscillate at its resonant frequency due to the bimetallic bimorph effect. The power consumption of the heating loop should not be higher than 0.1 W, otherwise damage may occur. Since the excitation is given as a percentage value in the software, the corresponding current *I* was measured and the power *P(heater)* was calculated:

(4) $$P\left(heater\right)=I^{2}*R\left(heat\right)\leq 0.1W$$

The resistance *R(heat)* of the heating loop is typically 28 Ohm. Consequently, the drive percentage has been set to a maximum of 20% to protect the cantilever heating loop.

### 2.5. Adaptation of the AFSEM^®^ for Measurements in Liquids

Dry state and vacuum measurements with the AFSEM^®^ scanner from GETec Microscopy (GETec Microscopy GmbH, Vienna, Austria) were performed without any changes to the system. For measurements in liquids, a new nose was designed and manufactured. This nose is shown in Figure 1b (left). The neck of the nose cone was extended for two reasons: (i) to allow the cantilever to be completely immersed in a sample chamber filled with liquid, and (ii) to increase the distance between the cantilever and the z-piezo. This ensures that the electronics are not damaged by liquid contact. Unlike the original nose, the piezo for acoustic excitation (2 × 2 × 2 mm P.I. Ceramics GmbH, Lederhose, Germany) was mounted through a hole from the top of the nose cone and positioned as close as possible to the sensor connector. For working in liquids, the supply voltage of the Wheatstone bridge was reduced from 2.048 to 0.51 V. To compensate for the lower electric potential of the signal, the gain has been increased by a factor of 4. Since the used scanner is designed as a tip-scanning AFM, a modified homemade sample stage was designed. The sample positioning in *x* and *y* was done by mechanical micromanipulators, while the *z*-axis approach was done by a fully automated z-stage with a 2″ piezo motor (Newport, Deckenpfronn, Germany). The scanner was mounted to the z-stage via a dove tail adapter and furthermore to a damped rod (Newport, Deckenpfronn, Germany). The control of the piezo motor is supported by the AFSEM^®^ control system.

### 2.6. Preparation of the Cell Samples

Platelets: Thin glass slides (35 × 20 mm, Fischer, Austria) were cleaned by sonification in ethanol and distilled water for 5 min each and dried under a gentle nitrogen stream. A drop of fresh blood was taken with the help of an insulin lancet and incubated for 1, 3 and 5 min respectively, on the freshly cleaned glass slides. After incubation, the cell samples were carefully rinsed with deionized water and gently fixed with 0.5% formaldehyde solution for 15 min. Finally, they were again rinsed with deionized water and dried under a gentle stream of nitrogen gas. The cell samples were stored at 4 °C and used within one week. For measurements in liquids, the samples were rehydrated with the corresponding liquids of interest.

Red blood cells: The glass cover slides were cleaned as described for platelet preparation or alternatively with isopropanol instead of ethanol. In case of gold carriers, 9 MHz QCM (quartz crystal microbalance) crystals (Renlux Crystal, Shenzhen, China) were incubated in basic piranha (3:1 ammonia water and hydrogen peroxide). After rinsing with deionized water (5 times), the cleaned gold surface and the glass slides were treated identically. One drop (300 µL) of a 0.01% poly-L-lysine (PLL) solution in deionized water was incubated for 30–90 min. Blood was freshly drawn from a vein into 9 mL EDTA blood collection tubes (Greiner, Kremsmünster, Austria) in a local hospital. Immediately thereafter, physiologically intact erythrocytes were separated by centrifugation. For this purpose, 4 drops of venous blood were diluted with isotonic phosphate buffered saline (PBS) (5 mM Na_2_HPO_4_, 150 mM NaCl, 200 µM ethylene glycol-bis(β-aminoethylether)-N,N,N′,N′tetraacetic acid EGTA, pH adjusted to 7.4 with HCl) and centrifuged with a microcentrifuge (Eppendorf mini spin) at 3800 rpm for 4 min. The supernatant was drained, and the pellet was resuspended in 1 mL isotonic PBS. This procedure was repeated three times. Finally, 1.5–3 µL of the pellet was dissolved in PBS. These purified erythrocytes were briefly incubated on the PLL-coated glass slides and immediately chemically fixed with 200 µL of 1% glutaraldehyde in PBS for 30–45 min. All wet samples were then washed three times with PBS for 5 min each and stored in PBS at 4 °C for a maximum of 2 days or used immediately for measurements in deionized water. Some of the samples were dried with a gentle stream of nitrogen and stored under argon for up to 4 weeks. A detailed protocol for the optimization of this procedure with regard to membrane orientation is out of the focus of this study and will be published elsewhere (Stainer et al., submitted).

### 2.7. Sample Preparations for Measurements in Blood, Blood Serum and Ink

After preparation of the erythrocyte sample and characterization under ambient conditions, the samples were rehydrated with freshly drawn blood or blood serum. For rehydration of erythrocyte samples with blood, the blood was diluted 1:8 with deionized water or used immediately without further dilution. For measurements in blood serum, the freshly drawn blood was centrifuged at 3800 rpm with a micro-centrifuge (Eppendorf mini spin) for 4 min to remove blood cells. The serum was taken from the centrifugation tube. These preparations were used for speed and noise measurements. For measurements in ink, a standard ink cartridge was opened, and the ink was diluted 1:1 with deionized water. All imaging solutions were used immediately after preparation.

### 2.8. Speed and Noise Measurements

Speed measurements were performed on a standard AFM calibration sample (HS-500MG AFM XYZ calibration standard). The imaging speed was increased stepwise from 30 to 750 µm s^−1^. Noise and speed measurements were performed using the very same PRSA-L300-F50-Si-PCB cantilever (SCL—Sensor.Tech., Vienna, Austria). The noise measurements were done in contact mode, where the noise was calculated from both the topography and the deflection image. Before the noise measurements from the topography signal were performed, a topographical image of the calibration grid with optimized feedback parameters was taken. To ensure that there is no change in topography during the noise measurement, the x, y scan size was reduced to a minimum (~1 pm). With these parameters, an image with 1 line per second and a resolution of 256 pixels was acquired. To determine the noise from the deflection signal, the feedback parameters were then reduced to a minimum, just high enough to prevent the cantilever from drifting away from the surface. These very low feedback parameters are intended to ensure that almost the entire signal is represented in the deflection as an error signal. Since we assume that there is no topographic change, the information of the deflection image reflects the noise of the system. Using these parameters, again, an image with the same resolution was taken. The deflection images were converted into distance units by determining the contact mode sensitivity. The corresponding images were quartered using Gwyddion, and the root mean square (RMS) values of the noise as well as the standard deviation were determined.

## 3. Results

### 3.1. System Integration and Adaptation

Replacing the conventional OBD-based detection system of an AFM with an optical-free, self-sensing detection system is an essential prerequisite for performing nano-medically relevant measurements in turbid fluids, such as human blood or milk. Piezo-resistors are a promising candidate as sensing elements. These are resistors in which their resistance value changes due to mechanical expansion or geometric changes. The integration of these piezo-resistors on a cantilever is usually realized as a Wheatstone bridge of four piezo-resistors. Two of them are placed in the bending area of the cantilever, while the other two are located on the cantilever chip. Before use, a voltage is applied to zero the Wheatstone bridge (Figure 1, left, highlighted in yellow), in order to compensate for differences in the resistor branches. This configuration with four piezo-resistors leads to an increased stability regarding thermal drift or self-heating compared to single sensor probes. In order to realize an all-electric (AE) Bio-AFM with a broad applicability, we have used commercially available self-sensing cantilevers (SSCs). We followed two approaches, the implementation of the self-sensing technology in an OBD-based AFM and the use of the AFSEM^®^ system, a SSC-based AFM, which we adapted for fluid measurements.

The optimal replacement of OBD of a commercial AFM by self-sensing cantilevers requires a design with a minimum of modifications to the existing AFM hardware and, ideally, without changing the controller software. As the AFM system, we chose a Keysight 5500 SPM2. In this system, the cantilevers are clamped by a simple mechanical spring. In contrast, an SSC requires electrical connections for the Wheatstone bridge as well as for thermal excitation, in addition to the mechanical clamping. Therefore, we have completely redesigned the nose cone (Figure 1a left).

The commercial SSCs used in this study are equipped with both a piezoresistive Wheatstone bridge for voltage-based deflection sensing, and a heating loop for thermal actuation. The implementation of these cantilevers in a commercial OBD setup requires a completely new nose design for the mounting of the self-sensing cantilever as well as the supply of the sensor and the readout of the deflection signal (Figure 1a). The new nose cone has been designed in close approximation to the original nose design, especially with regard to perfect fluid sealing and optical access via the AFM embedded CCD camera. A special printed circuit board was designed to mount and control the sensor. The flexible part of the circuit board is located on the outside of the new nose cone and is equipped with a connector for the cantilever PCB. The feed-through for the flexible part of the PCB is sealed to protect the internal electronics from fluid damage. To amplify the signal of the Wheatstone bridge as close as possible to its origin, the nose cone is equipped with an adjustable instrumentation amplifier. The deflection signal amplification can be adjusted depending on the measurement conditions (dry or liquid). The used sensor supply voltage of 2.048 V for imaging in dry conditions and 0.51 V for imaging in liquid environment is also realized in the nose cone. Since the space for the PCB is very limited and the smallest surface mounted device (SMD) standard had to be used, individual nose cones were manufactured for measurements in liquid and dry environment. Furthermore, the excitation pins of the AFM system were directly connected, either to the heating loop for thermal excitation or to the excitation piezo. For acoustic excitation, a piezo was embedded in the nose cone as close as possible to the cantilever. The Keysight 5500 SPM2 AFM used is designed in such a way that optical access is possible for both the OBD laser and CCD camera. Thus, all parts for all-electric readout have been placed outside the optical axis, which still allows the use of the CCD camera. The standard OBD scanner has 6 pins to cover the different operating modes with different nose cones (e.g., scanning tunneling microscopy and conductive AFM). The two pins, which are intended for cantilever excitation, are still used, while the other pins, which are not required for standard AFM imaging, are used to supply the new nose cone electronics with electricity/power and to transfer the amplified deflection signal to the subsequent hardware. This configuration ensured that no changes to the original scanner hardware were necessary.

In order to utilize the original OBD signal processing path for all-electric readout, the single deflection signal needs to be converted into an equivalent of the OBD signals. This must be achieved by mimicking the signal that the controller hardware typically receives from a four-segment photodiode. A second hardware has been developed for this purpose, including: (i) a signal conditioning hardware and (ii) an additional adjustable instrumentation amplifier. Here, the four signals of a four-segment photodiode are simulated from the single SSC deflection. The scheme of this signal processing is shown in Figure 1a (right), and the detailed description is given in the experimental part in Section 2.3. In short, the original deflection signal was inverted, and an offset was added to both the original and the inverted signal. Using the non-inverted deflection signal plus offset for the upper two segments and the inverted deflection signal plus offset for the two lower segments of the four-quadrant photodiode allows the SSC to be connected to the original controller hardware.

In contrast to the implementation of SSC in an OBD AFM, the upgrade of a commercial all-electric AFM (AE-AFM) for physiological measurements in (turbid) liquids requires an alternative approach. The used AFSEM^®^ was originally developed to perform measurements in vacuum (e.g., in the chamber of a SEM) or in air. Using the original configuration would damage the nose of the scanner by immersion in water, as open electrical connections would be short-circuited. As shown in Figure 1b (left), we have designed a nose with a significantly longer distance between cantilever and electronics (Figure 1b (left, arrow)). This, on the one hand, allows for safe measurements in liquids like none-conductive buffers and, on the other hand, the small nose shape allows experiments in Petri dishes and smaller chambers. In addition, the voltage supply for the Wheatstone bridge was reduced from 2.048 to 0.51 V to avoid the risk of undesired electrochemical reactions. The reduction of the supply voltage thus also causes a proportional change in signal intensity, resulting in a reduced signal-to-noise ratio. In contrast to the lower deflection signal due to the reduced supply voltage, the gain of the instrumentation amplifier has been changed from 100- to 400-fold. No further modifications of the system were necessary.

All these changes aimed to allow measurements in physiological fluids ranging from buffer and blood serum to pure blood or milk. We see a high potential for AE-AFM investigations in blood. Therefore, we have chosen blood compartments as the ideal feasibility test system to compare the performance of AE-AFM with OBD-based AFMs. For the direct comparison of the optical and the electrical readout, the ability to image simple calibration grids for all possible combinations was tested. These ranged from contact mode to three different excitations for intermittent contact mode with optical and electrical readout. As a result, we successfully performed measurements of a standard AFM grid using electrical readout in air, deionized water and freshly drawn blood. All these measurements were done in contact mode and in intermittent contact mode (acoustic as well as thermal excitation). Magnetic excitation for AE-AFM imaging was not realized due to a lack of magnetically coated SSCs. In addition, the same measurements were performed with optical beam deflection. Here, the thermal excitation was not shown as there was a lack of optical cantilever equipped with a heating loop. Data are shown in supplementary information (Supplementary Figure S1). It is obvious that OBD fails in optically non-transparent liquids. In contrast, AE-AFM allowed such investigations in turbid liquids, including blood and bovine milk.

### 3.2. Imaging of Biological Samples in Dry State

**Figure 2:**
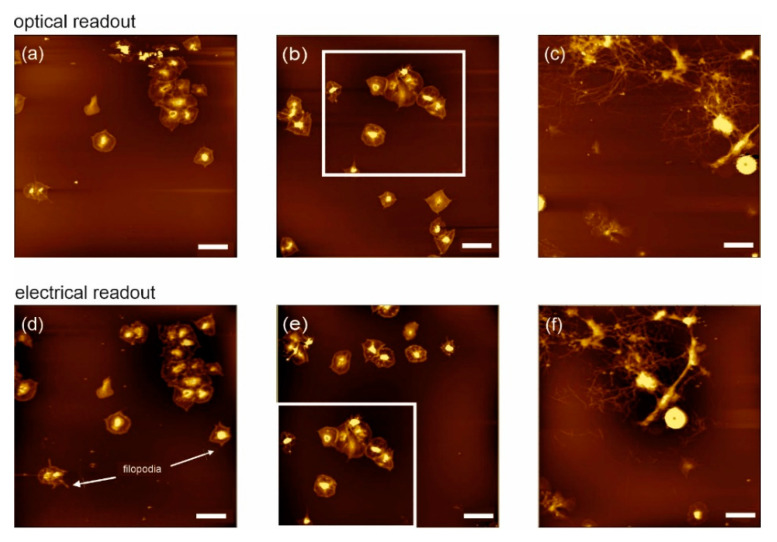
Comparison of optical and electrical readout on physiologically relevant samples in air: The upper part shows platelets after one (**a**), three (**b**) and five minutes (**c**) incubation, imaged with optical cantilevers and magnetic excitation. In comparison, the same sample positions were imaged with self-sensing cantilevers (**d**,**e**,**f**), yielding equal results (lower part). The scale bar is 10 µm for all images. Z-scale: (**a**,**d**): 191 nm, (**b**,**e**): 200 nm, (**c**,**f**): 900 nm.

Besides the comparison of different readout methods and excitation modes, a direct comparison of the image quality on highly relevant physiological samples is of interest. First, images of biologically relevant samples using the self-sensing cantilever readout on the OBD-based AFM were taken of human platelets in a dry state. Platelets are one of the major players in hemostasis. The conventional OBD-based AFM has been successfully used to study the activation mechanism at the single-cell [57] and single-molecule level using imaging, recognition imaging and single-molecule force spectroscopy [58]. The latter study was based on investigations on platelets that were partially fixed and dried in different activation states. In contrast to this earlier study, we focused here on fast (clot formation) processes that occur within the first minutes. Thus, fresh blood was incubated for 1, 3 or 5 min on a cleaned solid surface, followed by careful washing, gentle fixation and drying. In this study, however, the main purpose was to compare the performance of the AE-AFM with OBD readout in terms of topography. The images shown in Figure 2 are taken in air and show typical and expected platelet activation, including filopodia formation.

All three samples (1, 3 and 5 min) were initially imaged with a standard OBD readout system and a magnetically coated optical cantilever in intermittent contact mode (Figure 2a–c, respectively). After changing to our AE-AFM scanner nose and to a self-sensing cantilever, the same positions (guided by the AFM’s CCD camera) were imaged (Figure 2d–f). AE-AFM imaging was also performed in intermittent contact mode by means of acoustic excitation. Apart from the different levels of activation, it is shown that the imaging quality and capabilities are clearly comparable, and AE-AFM is perfectly suited for imaging biological samples. Even in the dry state, the advantages of using AE-AFM are obvious, ranging from (i) simplified usage due to the lack of optical adjustment, (ii) higher stability due to complete electrical readout [10] and (iii) the possibility of easy combination with other techniques (e.g., SEM).

### 3.3. Combined AFM–SEM Investigations and Measurements in Different Environments

**Figure 3:**
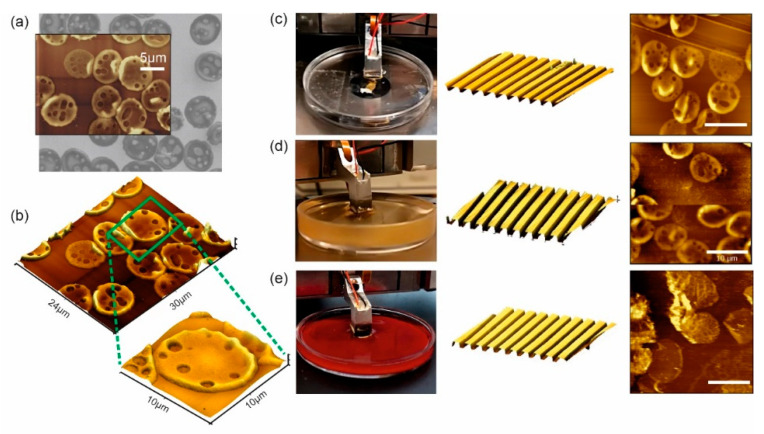
(left) Correlated atomic force microscopy—scanning electron microscopy (AFM—SEM): (**a**) Ultra-flat erythrocyte ghosts on gold were first recorded with the SEM and imaged with the AFSEM^®^ inside the SEM at the same position. (**b**) Overview and three-dimensional (3D) single erythrocyte AFM image at the same position. Images of ultra-flat erythrocyte ghost cells were taken in various liquids, such as deionized water (**c**), blood serum (**d**) and freshly drawn human blood (**e**). For each liquid, a proof-of-principle measurement of a calibration grid was performed ((**c**–**e**), middle), followed by imaging of erythrocyte ghost cells ((**c**–**e**), right). The grid in (**e**) was recorded in 1:8 diluted blood. Scale bars are 10 µm for AFM images in (**c**–**e**).

As a test system for combined simultaneous measurements with AFM and SEM, we have selected another physiologically highly relevant blood cell, the erythrocytes (RBC—red blood cells). In order to achieve optimal AFM and SEM resolution and to avoid artifacts caused by differences in cell stiffness, a new preparation of ultra-flat RBC ghosts was used. The erythrocytes were immobilized on a flat gold surface (e.g., a cleaned QCM), which allowed an adjustable membrane orientation. These samples were characterized by correlated AFM–SEM imaging. Areas of interest were determined by SEM imaging, followed by positioning of the SSC and acquisition of three-dimensional (3D) topographic images in the vacuum chamber of the SEM. The results are shown in Figure 3a,b. Most of the examined cells appeared extremely flat and represented their outer membrane. A detailed analysis showed that 35–55% of the gold surface was covered with ultra-flat erythrocyte ghost cells. Finally, we performed AE-AFM measurements in various fluids, ranging from deionized water to physiological media, such as blood serum, to human blood and bovine milk. As shown in Figure 3 (right side), we first started by imaging a calibration grid in deionized water, followed by the cell sample (Figure 3c). The same was done for blood serum (Figure 3d) and freshly drawn human blood (Figure 3e). On the left side in Figure 3c–e, the liquid nose of the AE-AFM is shown in the respective media. In the center, an AFM calibration grid measured in this liquid is presented. On the right side, a typical AFM image of ultra-flat erythrocyte ghosts in the corresponding medium is shown. It should be mentioned that the grid for blood was recorded in a 1:8 diluted blood:water solution to reduce contamination of the grid by products of blood clotting. In contrast to blood serum, native blood exhibits relatively rapid clotting phenomena induced by shear stress or compartments of the intrinsic or extrinsic coagulation cascade. Therefore, the ultra-flat RBC membranes in pure native blood showed some differences in their topographical appearance, which are strongly dependent on the clotting state of the blood (unaffected RBC images taken with 1:8 diluted blood are shown in the Supplementary Materials, Figure S1, lower right). Although artifacts can be caused, e.g., by undesired adhesion of platelets or other blood compartments to the sensor surface, technically, stable imaging over a sufficiently long time is possible. We were able to perform AFM imaging of such a preparation of ultra-flat RBC ghosts for more than four hours without any loss of performance in the readout system.

In summary, we were able to show comparable results on both systems, the adapted OBD AFM and the AFSEM^®^ adapted for working in liquids (AE-AFM). Furthermore, combined SEM and AFM images of the same sample position in vacuum were shown. More importantly, measurements of blood compartments such as platelets or erythrocytes are possible and were shown—for the first time ever—in their real native environment (i.e., undiluted human blood). Nevertheless, it must be mentioned that long-term experiments in native blood require further physiological and technical optimizations with respect to imaging conditions, ranging from adjustable blood flow to controlled sedimentation and passivation of the cantilever surface, but the technical limitations in general have now been demonstrably overcome.

### 3.4. Detailed System Characterization

In addition to the technical realization of all-electric AFM imaging, including first measurements of cell samples in turbid liquids like blood, we investigated and compared the imaging performance in different media, like deionized water, ink, blood serum and pure blood. A key parameter is the z-noise of the system. This z-noise is composed of three main parts: noise caused by the cantilever movement, noise caused by the measuring principle and noise of the readout electronics [15]. In both readout methods, OBD or SSC, the combination of these three noise sources yield the limit for the minimum detectable deflection. The latter equals to the root mean square (RMS) voltage noise [15]. The measured z-noise can be strongly influenced by external noise sources such as acoustic noise, mechanically transmitted vibrations or electronic noise. Thus, it is necessary to minimize external sources of interference before determination of the z-noise caused by the setup itself. To optimize the noise level, measurements were performed with a mechanically fixed sample stage and minimized mechanical loop in a closed glove box on a passively damped table. To exclude deviation caused by different cantilevers, all noise measurements were always performed with the same cantilever. The RMS AFM imaging noise was characterized by so-called two-dimensional (2D) noise images in contact mode. For noise determination, we used two different approaches, (i) the determination of the noise from a topographical image, and (ii) from the deflection image. Before determining the z-noise from the topography, an image was taken from a calibration grid with optimized feedback parameters. In order to obtain a 2D noise image without topographic changes, the x, y scan size was set to a minimum value. To determine the noise from the deflection image, the feedback parameters were minimized to avoid any shift of information from the deflection/error into the topographical image. To convert the voltage signal into a length measurement, the contact mode sensitivity was determined.

As a reference, the system z-noise was determined in dry conditions (2.91 Å (topography method)). The noise values in deionized water, diluted ink, blood serum, as well as in diluted and pure human blood, are shown in Table 1. In liquid, the z-noise is somewhat higher, whereby, for example, a value of 3.52 Å (topography method) was measured for blood. The noise values from the deflection images are slightly lower compared to those from the topography method but show a similar trend (2.63 Å for air, 3.1 Å for blood). Compared to previous noise measurements with self-sensing cantilevers of this type, focusing on the minimal reachable z-noise [15], we are about a factor 3 higher at these typical measuring conditions. This may be caused by the additional mechanical loop of the highly flexible AE-AFM z-stage and the different setup design (tip scanning). We could show that changing from dry state to different solutions, including highly turbid blood, yield very minor increases in the z-noise. In summary, it can be said that even with measurements in pure blood, a sufficient z-resolution for physiological measurements (i.e., 0.30 ± 0.15 nm) can be achieved.

**Figure 4:**
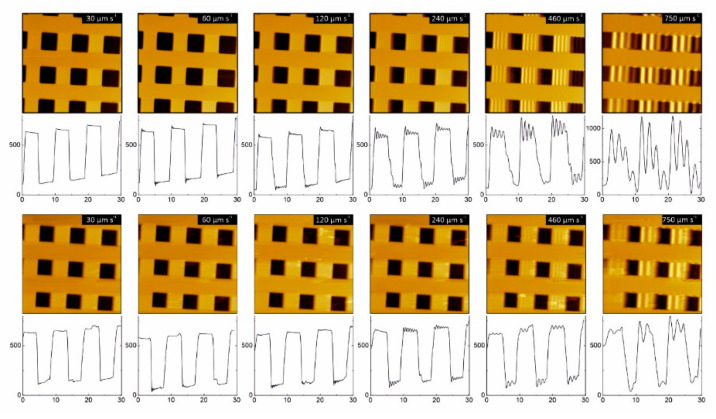
Comparison of speed performance in different media. The upper part shows images acquired in deionized water, and the lower part in pure human blood. The speed has been increased from 30 µm/s (1 s/line) to 750 µm/s (0.04 s/line). The corresponding horizontal cross-sections have been extracted from the very same position in the middle row of the image. Measurements have been performed on a HS-500MG AFM XYZ calibration standard. *x*, *y* scan range is 30 µm for all images.

We further investigated the influence of the imaging speed, especially in turbid liquids, using our optimized AE-Bio AFM. Although the used AE-AFM was not developed as a high-speed AFM system, we investigated and analyzed the influence of different fluids on the imaging performance of moderate and higher scanning rates. For all these measurements, the same PRSA-L300-F50-Si-CB 300 × 100 cantilever was used to allow an unbiased comparison. As a sample, we used an AFM XYZ calibration grating and a scan size of 30 µm. The tip velocity was varied from 30 to 750 µm s^−1^. First, reference measurements were taken in contact mode in air, followed by the different liquids, like deionized water, diluted ink, serum, as well as diluted and pure human blood. Figure 4 shows a comparison of the performance in deionized water with the most challenging environmental condition, pure fresh human blood.

As can be seen from the topography as well as from the corresponding cross-section (Figure 4, upper part), imaging in deionized water works fine at standard speed (30 µm s^−1^) and up to an imaging speed of about 60 µm s^−1^. At a speed of 60 µm s^−1^, the first artifacts appear and can be seen in the cross-section (pits of the calibration grid, Figure 4, upper part, second image). With a further stepwise increase of the speed to 240 µm s^−1^, the feedback artifacts increase and are clearly visible in the topography. Especially when using imaging velocities of 750 µm s^−1^ or higher, the quality of the topographical images is strongly distorted. The same results are also obtained in pure blood (Figure 4, lower part), showing first feedback artefacts at similar scanning speed. Speed measurements at all other environmental conditions are shown in Supplementary Figure S2. By comparing the speed measurements at all different imaging conditions, it becomes clear that the scanning speed is not significantly influenced by measuring in (turbid) liquids. In summary, we proved that the z-noise in blood increases by about 35% compared to deionized water and no limitations in scanning speed appear by measuring in turbid viscous blood compared to the reference measurement at dry conditions. In contrast, we can conclude from the images recorded in Figure 4 that the speed performance in blood is even slightly superior to the measurements in deionized water. The scan speed is determined by the spring constant of the cantilever, its effective mass, the damping constant of the cantilever in the surrounding medium and the stiffness of the sample [59]. The viscosity of human blood depends on different factors, especially from the shear rate. It is in the range of 3.26 ± 0.43 mPa s at a shear rate of 100 s^−1^ to 5.46 ± 0.84 mPa s at a shear rate of 1 s^−1^. In contrast, viscosity of water at 20 °C is 1 mPa s [60]. In blood, the damping constant and the effective mass of the cantilever are also increased. At our moderate imaging velocities, the viscous drag effect still allows correct data but the increased damping constant and effective mass explain the minor shift of the imaging speed limit compared to measurements in deionized water.

## 4. Discussion

The technical challenge to perform biological AFM imaging in turbid liquids is now solved by combining commercially available and sufficiently soft SSCs with upgrades in AFM design and electronics. Although we are able—for the first time ever—to present images of blood cells in highly turbid fresh human blood, the all-electric AFM as a tool for in-situ investigation of clot formation is still at a very early stage. As mentioned above, mimicking arterial blood flow requires precise control of physical, biochemical and medical parameters. In addition, anti-adhesive coatings of the surrounding surfaces may be required. In any case, we are convinced that all these requirements are feasible, making the AE-AFM to a key tool in hemostatic research. By constructing a suitable sensor nose design, AE-AFM also has the potential to observe passivation and undesired coagulation in stents and can therefore play a crucial role in the development of stents and stent coatings. In addition, our biosensing all-electric AFM technically allows further characterization methods, including high-resolution elasticity mapping, gaining mechanical data, nano-indentation and functional investigation. Simultaneous topography and recognition imaging is currently not implemented but is in the planning stage. In contrast to conventional OBD-based AFM, our AE-AFM developments simplify the usage of cantilever arrays for the investigation of biological samples (e.g., in blood). This may open new great opportunities like multi-information topography and recognition imaging using different bio-functionalized cantilever on the same sample in a parallel manner. Finally, it should not be overlooked that AE-AFM measurements could be used as a powerful (nano-) investigation tool in completely different areas, such as industrial applications (e.g., milk tank inspection) or biofilm formation in optically non-transparent liquids. We believe that SSC-based bio-AFMs will become a promising tool, especially in the biological/physiological/medical sciences.

## Figures and Tables

**Table 1:** Comparison of z-noise values recorded in different media.

Noise in Different Media		Dry State [Å]	Deionized Water [Å]	Diluted Ink [Å]	Blood Sera [Å]	Diluted Blood [Å]	Pure Blood [Å]
Topography	RMS *	2.91	3.64	3.36	3.62	3.03	3.52
	SD ^#^	0.024	0.02	0.043	0.15	0.077	0.069
Deflection	RMS *	2.63	3.00	2.67	2.50	2.31	3.10
	SD ^#^	0.026	0.042	0.023	0.07	0.026	0.15

* root mean square; ^#^ standard deviation.

## Supplementary Materials

The following are available online at https://www.mdpi.com/1424-8220/20/13/3715/s1, Figure S1: Comparison of electrical and optical readout, Figure S2: Comparison of AE-AFM speed measurements at different environmental conditions.
