# Supplementary material for: Atomic Force Microscopy Imaging in Turbid Liquids: A Promising Tool in Nanomedicine
Source: Sensors (Basel). 2020 Jul 2;20(13):3715. doi: 10.3390/s20133715 (PMC7374447; doi:10.3390/s20133715)
Supplement: Supplementary file 1 [file sensors-20-03715-s001.pdf]

Figure S1

		optical readout			electrical readout		
		ambient condition	deionized water	turbid liquids	ambient condition	deionized water	turbid liquids
contact mode		✓	✓	✗	✓	✓	✓
intermitttent contact mode	acoustic excitaion	✓	✓	✗	✓	✓	✓
	magentic excitation	✓	✓	✗	✓	✓	✓
	thermal excitation	✓	✓	✗	✓	✓	✓

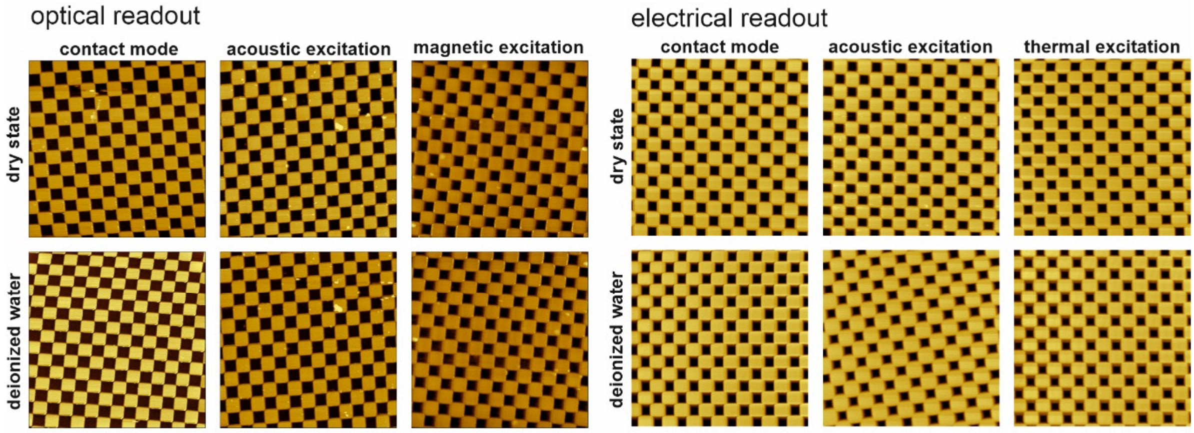

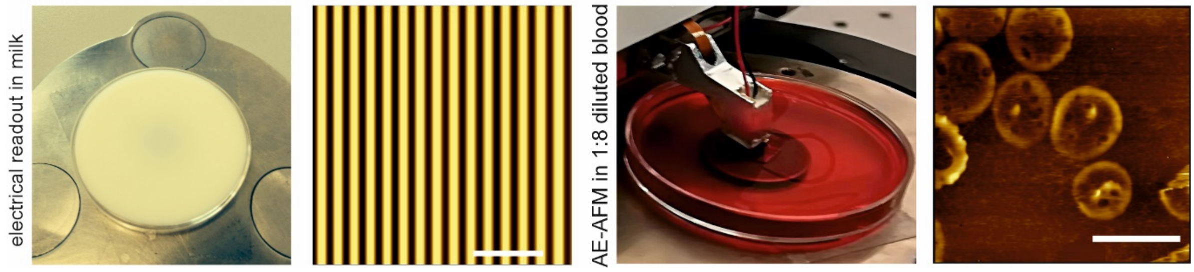

Figure S1: Comparison of electrical and optical readout. Upper: Table of realized (green) and possible, not realized (grey) and not realizable combinations (red). Middle: Corresponding AFM grid images (TGX1 calibration grating, image size is 20 µm) of all realized combinations. Lower left: Sample chamber and (TGZ2 calibration grating, x, y scale bar is 10 µm) imaged in bovine milk. Lower right: Sample chamber and ultra-flat erythrocyte ghosts imaged in 1:8 (blood:water) diluted human blood.

Figure S2

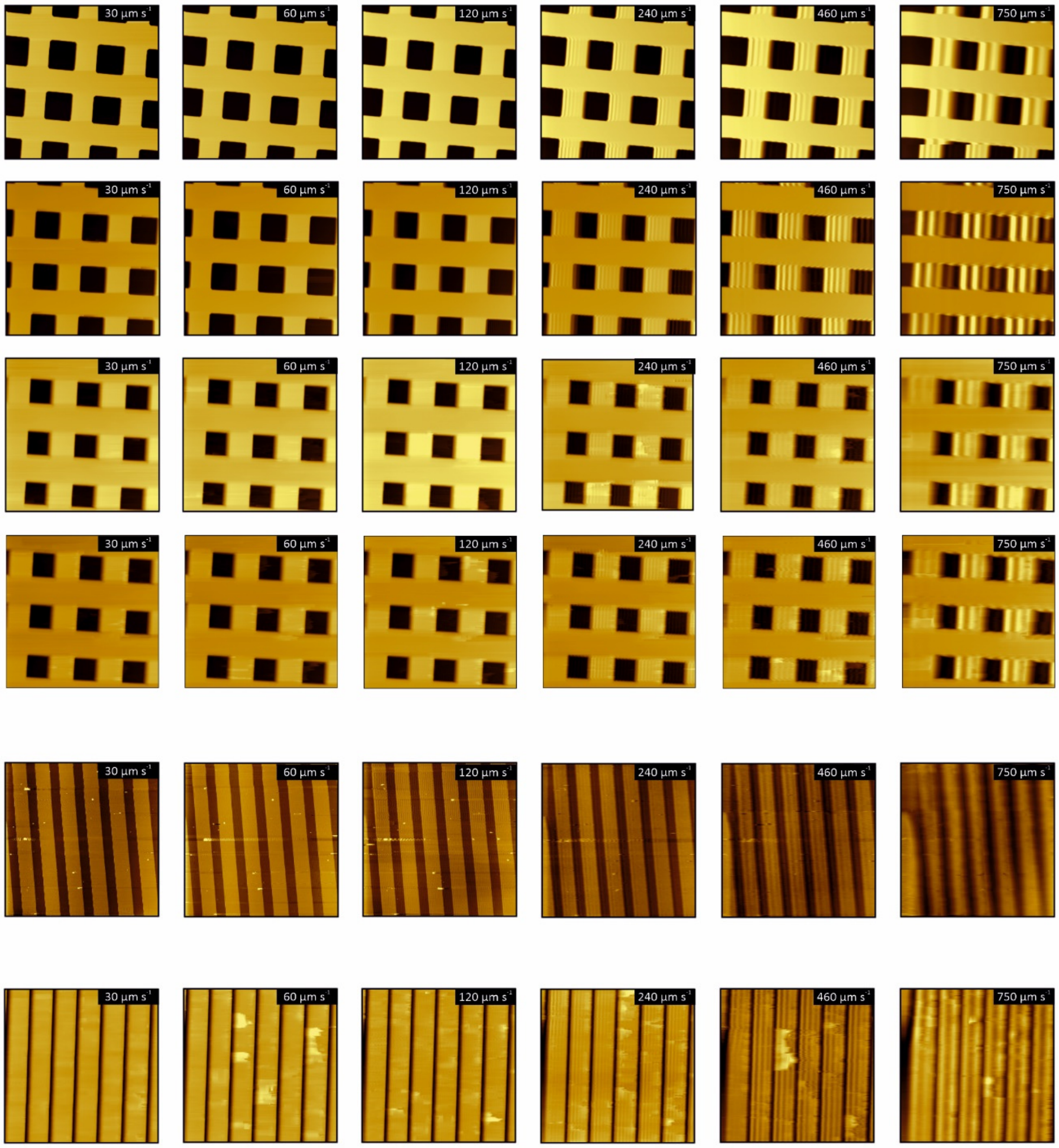

Figure S2: Comparison of AE-AFM speed measurements at different environmental conditions. First line: air, second line: deionized water, third line: 1:3 diluted blood (blood:blood serum), fourth line: pure human blood, fifth line: ink, sixth line: blood serum. Image size 30 µm, z-scale 800 nm, grid lines 1–4 and 6: HS-500MG AFM XYZ calibration standard, grind line 5: HS-20MG AFM XYZ calibration standard, z-scale 60 nm.
